# The diversity of Odonata and their endophytic ovipositions from the Upper Oligocene Fossillagerstätte of Rott (Rhineland, Germany)

**DOI:** 10.3897/zookeys.130.1441

**Published:** 2011-09-24

**Authors:** Julián F. Petrulevičius, Torsten Wappler, André Nel, Jes Rust

**Affiliations:** 1 CONICET-Museo de La Plata-UNLP, División Paleozoología Invertebrados, Paseo del Bosque, s/n, 1900 La Plata, Argentina; 2 Steinmann Institut für Geologie, Mineralogie, Paläontologie, Universität Bonn, Nussallee 8, 53115 Bonn, Germany; 3 CNRS UMR 7205, Muséum National d’Histoire Naturelle, CP 50, Entomologie, 45 rue Buffon, F-75005 Paris, France

**Keywords:** Insect reproduction, plant-insect interactions, Zygoptera, Odonata, palaeoecology, Oligocene, Rott

## Abstract

A commented list of fossil Odonata from the Oligocene outcrop of Rott is given, together with descriptions of new traces of oviposition in plant tissues, very similar to ichnotaxa already known from the early Eocene Laguna del Hunco floras of Patagonia. The joint presences of odonatan larvae and traces of oviposition demonstrate the autochthony of these insects in the palaeolake of Rott, confirming the existence of a diverse and abundant aquatic entomofauna, a situation strikingly different to that in the contemporaneous Oligocene palaeolake of Céreste (France).

## Introduction

Since the first half of the 19th century the Oligocene lake deposits of Rott are well known for their high diversity of fossil insects. Until the middle of the 20th century about 630 species have been described (e.g., Lutz 1996). With almost 11.5 % of insect fossils, the odonates are quite common, mainly represented by their nymphs, but also by a variety of adult specimens with at least 11 species as well as by oviposition traces on fossil plant material. Females of most odonate species lay their eggs into plant substrata. Endophytic oviposition in these insects is performed by means of the ovipositor valves, which penetrate and cut substrate tissues inserting eggs in the prepared slits (e.g., Wesenberg-Lund 1943). Damselflies and dragonflies can use both live and rotten plants as oviposition substrates. The Cenozoic record of odonate endophytic oviposition is rather numerous and of modern aspect. Previous records have been recently summarized in Sarzetti et al. (2009). However, (Hellmund and Hellmund (1991, 1996c, 2002a) were the first to described two scar patterns for the Rott fossil site. They recognized the most common “Coenagrionid Type”, for which the arcuate and zigzag arrangements are the modal configurations, and the less common “Lestid Type”, in which the files of paired egg scars follow the stronger veins. The arcuate/linear and zigzag arrangements of ovipositions could also be attributed to the damsel-dragonflies (represented in Recent by the relic family Epiophlebiidae), or to aeshnid dragonflies (for explanation see below). Furthermore, the locality is characterized to have a rather high diversity of body fossils of Odonata with at least 11 species based on adults and nymphs (*vide infra*).

The Rott fossil locality, lying between the town of Hennef and the Pleisbach River in the northern Siebengebirge close to the city of Bonn, is known for its abundance of exceptionally well-preserved fossil plants and animals (e.g., von Koenigswald 1996) as well as abundant insect damage on the bulk flora (Wappler 2010). Due to early mining activities, its stratigraphy is well-known (e.g., von Dechen 1884; Kaiser 1897; Laspeyres 1900; Wilkens 1927), and the fossiliferous layers were already discovered in several mines by the 19th century. 200 plant taxa and 35 vertebrate taxa, as well as over 600 insect taxa have already been described (numerous works of Statz in the years 1930–1952), although most of these descriptions are in need of revision, being nearly 150 to 70 years old (Lutz 1996; Fikáček et al. 2010). No new finds from Rott are expected, since only overgrown and weathered small dumps of rocks remain there.

The earliest notes on plant remains from Rott can be found in Weber (1852). Leaves that could be morphologically classified and identified were especially featured in this comprehensive study, as was the fashion at the time. In the following years, he collaborated with Philipp Wessel (1855) and they expanded the faunal inventory. Between 1937 and 1948, the leaf flora was updated, especially by Herrmann Weyland – this involved not only the original studies by Weber and Wessel, but also new material, particularly from the so called Statz collection.

Over 120 studies about the fossil insects from Rott have been published, making it as relevant as other famous Cenozoic localities, like Enspel and Céreste, but no taphonomic study of the Rott insects has been done yet. Besides small correspondences about specific taxa (e.g., Statz 1931, 1936, 1940), the extensive revisions of single dipteran groups (e.g., Statz 1943, 1944a,b,c) stimulated further research on the insect fauna of Rott (see Fikáček et al. 2010). The dominance of different *Plecia* species (Diptera: Bibionidae) and the large diversity of isopterans is indicative for a warm, paratropical climate (Collomb et al. 2008); this is also supported by the palaeobotanical finds.

According to Lutz (1996), the Rott insect taxa are percentually distributed as follows: Diptera (26%), Coleoptera (24%), Hymenoptera (19%), and Orthoptera (18%). In contrast to similar localities (e.g., Enspel, Messel, Eckfeld, Céreste or Aix-en-Provence), aquatic insect larvae are quite common; sometimes they have been even found en masse (Statz 1938, 1950). Besides heteropterans and odonates, aquatic Coleoptera represent around 25% of the finds.

Nevertheless, additional screening of private and historical collections reveals new and undescribed plant material. Based on distinctive morphologies and damage patterns of elongate, ovoid, lens-, or teardrop-shaped scars in the leaves, we could assign these insect damages to the ichnogenus *Paleoovoidus*, consisting of the ichnospecies, *Paleoovoidus rectus*, *Paleoovoidus arcuatus* and *Paleoovoidus bifurcatus* (Table 1, Figs 11–23). The data support recent palaeobiological studies of insect damage on fossil plants and thus can provide valuable information about insect diversity, ecological interactions, and evolutionary adaptation (e.g., Labandeira 2002: Fig. 2).

**Table 1.** Odonatan endophytic oviposition from the Upper Oligocene of Rott, Germany. §modified from Hellmund and Hellmund (1991) #*sensu* Sarzetti et al. (2009)

Collection no	patterns of ovipositional plant damage§	Host plant	Ichnospecies#	Reference
GPIBo Rott HELL-854	“Coenagrionid/damsel-dragonfly-Type”	*Apocynophyllum* sp.	*Paleoovoidus arcuatus*	Hellmund 1986, 1987, 1988; Hellmund and Hellmund 1991, 1996a, b
GPIBo Rott HELL-852	“Coenagrionid/damsel-dragonfly-Type”	unknown	*Paleoovoidus arcuatus*	Hellmund and Hellmund 1991
GPIBo Rott HELL-851a+b	“Coenagrionid/damsel-dragonfly-Type”	unknown	*Paleoovoidus arcuatus*	Hellmund and Hellmund 1991
SMNS 22147	“Coenagrionid/damsel-dragonfly-Type”	unknown	*Paleoovoidus arcuatus*	Hellmund and Hellmund 1991
SMNS 22148	“Coenagrionid/damsel-dragonfly-Type”	? *Salvinia* sp.	*Paleoovoidus arcuatus*	Hellmund and Hellmund 1991
SMNS 22149	“Coenagrionid/damsel-dragonfly-Type”	unknown	*Paleoovoidus arcuatus*	Hellmund and Hellmund 1991; Hellmund and Hellmund 2002a
Slg. Hellmund, ohne Nr.	“Coenagrionid/damsel-dragonfly-Type”	*Daphnogene cinnamomifolia*	*Paleoovoidus arcuatus*	Hellmund and Hellmund 1993
GPIBo_Ro_10982	“Coenagrionid/damsel-dragonfly-Type”	*Laurophyllum pseudoprinceps*	*Paleoovoidus arcuatus*	This study
GPIBo_Ro_10355	“Coenagrionid/damsel-dragonfly-Type”	unknown	*Paleoovoidus arcuatus*	This study
GPIBo_Ro_11887	“Coenagrionid/damsel-dragonfly-Type”	unknown	*Paleoovoidus arcuatus*	This study
HW_Ro_2.8	“Coenagrionid/damsel-dragonfly-Type”	*Sideroxylon salicites*	*Paleoovoidus rectus* / *Paleoovoidus arcuatus*	This study
GPIBo ohne Nr.	“Lestid-Type”	*Daphnogene cinnamomifolia*	*Paleoovoidus bifurcatus*	Hellmund 1988
SMNS 22144	“Lestid-Type”	*Daphnogene cinnamomifolia*	*Paleoovoidus bifurcatus*	Hellmund and Hellmund 1991; Hellmund and Hellmund 1996b; Hellmund and Hellmund 2002a
SMNS 22145	“Lestid-Type”	*Daphnogene cinnamomifolia*	*Paleoovoidus bifurcatus*	Hellmund and Hellmund 1991; Hellmund and Hellmund 1996b
SMNS 22146	“Lestid-Type”	*Daphnogene cinnamomifolia*	*Paleoovoidus bifurcatus*	Hellmund and Hellmund 1991; Hellmund and Hellmund 1996b
HW_Ro_58.2	“Lestid-Type”	*Daphnogene cinnamomifolia*	*Paleoovoidus bifurcatus*	This study

## Material and methods

### Material

The fossils examined in the present work are from a number of collections. Fossil leaves and insects from the Upper Oligocene of Rott are housed in the Steinmann Institute, University of Bonn (represented by the Statz Collection, Kastenholz Collection, the Geological–Palaeontological–Institute Collection [GPIBo] at Bonn); in the Collection of Heinz Winterscheid, Cologne (HW-Ro); the Staatliches Museum für Naturkunde (SMNS), Stuttgart, all in Germany; plus a great part of original collection of Statz with many types stored at the Natural History Museum of Los Angeles County (Sphon 1973).By far the most important collection of fossils from Rott was accumulated between 1930–1940 by private collectors, in particular Georg Statz and Anton Kastenholz. Heinz Winterscheid and Meinolf Hellmund also collected valuable material over the last 30 years. Fossils were examined in dry conditions using a binocular microscope, and photographed using a Nikon Coolpix 4500 digital camera, free or attached to the ocular piece of the microscope. The nomenclature of the dragonfly wing venation is based on the interpretations of Riek and Kukalová-Peck (1984), amended by Nel et al.(1993) and Bechly (1996).

## Geological Setting

### Locality and stratigraphy

The fossiliferous sediments, also known as the “Sapropelite- and Diatomite-Layers”, consist of alternating sapropelites, diatomites, radiolarites, bituminous clays and lignite layers reaching a height of 3–5 m. They partly rest on the weathered tuffs (e.g., Mörs 1995). Absolute dates are only available from volcanic rocks from the Siebengebirge (Todt and Lippolt 1980; Vieten et al. 1988; Wijbrans et al. 1995); in the central Siebengebirge, they have been dated to 26.4–23.0 Ma (Upper Oligocene, Chattian sensu Gradstein and Ogg 2004). Chronostratigraphically, the sediments belong to the uppermost Upper Oligocene, biostratigraphically (according to the mammal fossils) to Zone MP 30 (Mörs 1995); MP 30 ranges from Subchron C7An to the top of Subchron C6Cn.2r (see Agusti et al. 2001). Therefore, the absolute age of the Rott locality is 24–23 Ma (e.g., Kempf et al. 1997; Böhme 2003). The estimated mean annual temperature (MAT) in this area at the accumulation time of lake sediments was 17.5±1.2°C, based on leaf margin analysis (Winterscheid 2006a, b).

### Diversity of body fossils of Odonata

**Figures 1–6.**
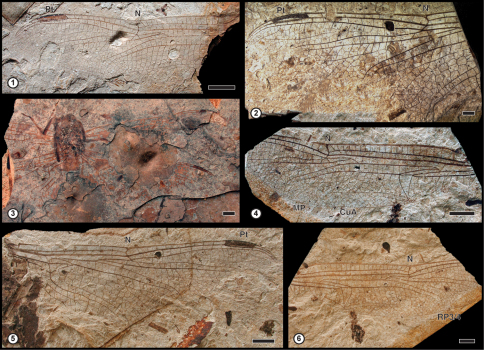
*Paleotramea cellulosa* (Hagen, 1863) from the Upper Oligocene Sapropelite- and Diatomite-Layers of Rott **1** Photograph of GPIBo A-626 **2** Photograph of GPIBo A-637a **3** Photograph of GPIBo A-624 **4** Photograph of GPIBo A-636b **5** Photograph of GPIBo Ro-2032 **6** Photograph of GPIBo A-636 a. Abbreviations: **N** – nodus; **Pt** – pterostigma; **RP3/4** – posterior radius; **MP** – posterior media; **CuA** – anterior cubitus. Scale bars represent 5 mm.

**Figure 7.**
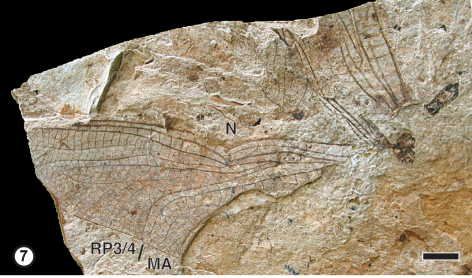
Libellulidae species A from the Upper Oligocene Sapropelite- and Diatomite-Layers of Rott.7 Photograph of GPIBo Ro-37a. Abbreviations: **N**– nodus; **RP3/4**– posterior radius; **MA**– anterior media. Scale bar represent 5 mm.

**Figures 8–10.**
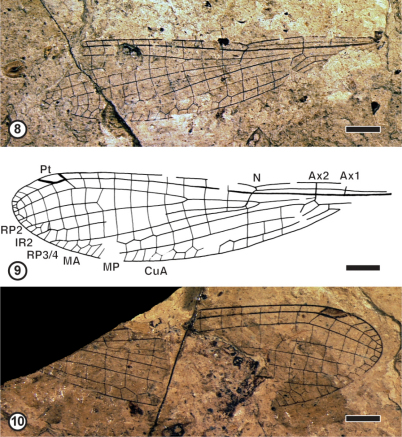
Coenagrionidae Kirby, 1890, subfamily and genus undetermined, species A, specimen; coll. Kastenholz **8** Photograph of GPIBo KH-1a **9** Camera lucida drawing GPIBo KH-1a **10** Photograph of GPIBo KH-1b. Abbreviations: **Ax** – costal braces; **N** – nodus; **Pt** – pterostigma; **RP** – posterior radius; **IR** – intercalated vein; **MA** – anterior media; **MP** – posterior media; **CuA** – anterior cubitus. Scale bars represent 2 mm.

The site of Rott is characterized to have a nice diversity of body fossils of Odonata with at least 11 species based on adults and nymphs. The Libellulidae are the most frequently encountered both nymphs and adults. This group has four species, the Libellulidae: Trameinii *Paleotramea cellulosa* (Hagen, 1863) (Nel and Paicheler 1993b), represented by adults (see Figs 1–6), and other three indeterminate species. Hagen (1863) attributed several nymphs to the genus *Libellula* under the names *Libellula ceres* Hagen, 1863 and *Libellula cassandra* Hagen, 1863, but this generic determination is uncertain, and they have to be considered as Libelluloidea *incertae sedis*. A new libellulid species A is described herein by an adult specimen (Fig. 7). *Aeshna dido* Hagen, 1963, based on a nymph, can be considered as an Aeshnidae *incertae sedis*. Other undeterminate Anisoptera (Nel and Paicheler 1994b) complete the odonatan diversity: *Ictinogomphus (Ictinus) fur* Fraser, 1957, based on an adult specimen, and five ‘species’ of damselflies. These are: one Lestidae, *Lestes statzi* Schmidt, 1958 (Nel and Paicheler 1994b) represented by adults; an adult of Coenagrionidae *incertae sedis* described herein (Figs 8–10); and several nymphs, viz. *Agrion icarus* Hagen, 1863 (Coenagrionoidea *incertae sedis*, after Nel and Paicheler 1993a), and *Coenagrion (Agrion) thais* Hagen, 1863 and *Coenagrion (Agrion) mysis* (both in Zygoptera incertae sedis, after Nel and Paicheler 1993a). In Rott there is also present the basal epiproctophoran family Sieblosiidae, with one described species, *Oligolestes grandis* (Statz, 1936) (Statz 1936; Schmidt 1958; Nel and Paicheler 1994a).

### Diversity of ichnofossils

Fossil endophytic oviposition of insects on plant organs are among the rarest, but also the most revealing traces of plant-insect associations in the geological record (e.g., Labandeira 2002; Béthoux et al. 2004; Vasilenko and Rasnitsyn 2007) but only late Cretaceous and Cenozoic oviposition scars can be reliably assigned to several insect groups, mainly Odonata (e.g., Hellmund and Hellmund 1991, 1993, 1996a, b, c, 2002a; Labandeira 2002; Labandeira et al. 2002; Vasilenko and Rasnitsyn 2007; Sarzetti et al. 2009). (Hellmund (1986, 1987, 1988) was the first to mention oviposition scar pattern from the Upper Oligocene of Rott but he was only able to list them as undetermined oviposition scars at that time. Later, the specimens have been transferred by (Hellmund and Hellmund (1991, 1996c, 2002a) to the Odonata, but they were described without any ichnotaxonomic analyses. Thus, they referred the scars to the most common “Coenagrionid Type”, for which the arcuate and zigzag arrangements are the modal configurations, and the less common “Lestid Type”, in which the files of paired oviposition scars follow the stronger veins and attribute them to oviposition habits. The linear/arcuate zigzag modal was referred to the coenagrionids but in fact could be found also in Anisoptera (Aeshnidae) and probably also in relatively more basal extinct Epiproctophora, as it is present in Recent Epiophlebiidae (Shimura 2005; Matushkina 2007). One important fact is that some Coenagrionidae, the Aeshnidae and Epiophlebidae lay their eggs in stems of aquatic plants (Shimura 2005; Matushkina 2007) and not in leaves like ichnofossils in present study. Taking apart the problem of the stem/leaf oviposition, for the moment there are not characters to distinguish the analogue (linear/arcuate zigzag modal of) oviposition of these groups to make accurate attributions in the fossil record. Possible characters could be the resulting ‘drawing’ (letters V, W, Z), angle and shape between the rows. In coenagrionids the angle could be open near 90° and the rows smoothly changing their direction (*vide* Sarzetti et al. 2009), in the Epiophlebiidae the angle is less opened, less than 45°, rows of eggs are more or less parallel, and change their directions abruptly (*vide* Shimura 2005). The zigzag modal of aeshnids is quite different with parallel rows each other resulting in a Z without its middle portion. Other interesting feature to take into account is the length of the egg laying. epiophlebiid females produce long egg laying in zigzag pattern (*vide* Shimura 2005) as described here for Rott (Fig. 19) and also present in Laguna del Hunco, Eocene of Argentina (Sarazetti et al. 2009: 441, fig. 5.6).

At Rott the distinguished damage occurred at least on five different host plants (Apocynaceae, Salviniaceae, Rhamnaceae, Lauraceae, Sapotaceae Hellmund 1986, 1988; present study), whereas lauraceous leaves (*Cinnamomum*- and *Laurophyllum*-type leaves) showing a marked preference for oviposition. However, the species with linear/arcuate zigzag ovipositions from the same locality seem to have been less selective, and even deposited their eggs in floating leaves of aquatic ferns (Table 1; Hellmund and Hellmund 1991). A broad spectrum of plant hosts for this ichnofossil is observed also in the Eocene of Laguna del Hunco, Argentina (Sarzetti et al. 2009; Petrulevičius pers. obs.). This fact also could be related that this trace seems to be made by different groups of Odonata as the coenagrionids and damsel-dragonflies as discussed above.

Contrary to previous statements (e.g., Müller 1976) leaf damage produced by ovipositors is relatively abundant throughout the Cenozoic (comp. Sarzetti et al. 2009).The percent of leaves with damage produced by ovipositors is significantly higher at Rott than at any other Oligocene site (unpublished data), whereas 2.78% of the damaged leaves show some kind of endophytic oviposition. They are especially rare at Aix-en-Provence, lacustrine outcrop of similar age (Nel pers. obs.). For the investigated Cenozoic localities oviposition frequency is highest at Messel, where over 3.3% of leaves show endophytic oviposition (Wappler pers. obs.).

## Systematic palaeontology

### Suborder Anisoptera Selys, 1854

**Family Libellulidae Leach, 1815**

**Libellulidae undetermined**

#### Libellula cassandra

Hagen, 1863

##### Remarks.

This species is based on larval stages. Hagen (1863) placed the specimens in the recent genus *Libellula* but the morphological characters are insufficient for this attribution. We have to consider it as an undetermined Libelluloidea, following Nel and Paicheler (1993b).

#### Libellula ceres

Hagen, 1863

##### Remarks.

Same remarks as for Same remarks as for *Libellula cassandra*.

#### Libellulidae species A

Fig. 7

##### Material examined.

GPIBo Ro-37a from the locality of Rott (Upper Oligocene, Sapropelite- and Diatomite-Layers): three fragmentary preserved wings; body and appendages missing.

##### Description.

Three wings articulated but wrinkled. Only antero-apical part of one wing is possible to be described. Nine postnodals preserved, not aligned with 10 postsubnodals. Pterostigma covering three cells. Nine posterostigmal veins.Pseudo-IR1 long born beneath posterior part of pterostigma and covering 11 cells. IR1 almost straight connected to pseudo-IR1. RP2 slightly curved. IR2 ending at more than three cells from RP2.Rspl almost straight but ending on IR2 distally, with two rows of cells between it and IR2.

##### Remarks.

The shape of Rspl ending on IR2 and those of IR1 and pseudo-IR1, together with the weak pterostigmal brace are typical of Libellulidae. The postnodals and postsubnodals not aligned together with the broad area between RP1 and RP2 suggest possible affinities with the Trameinae. Nevertheless, this fossil differs from *Paleotramea cellulosa* from the same outcrop in the presence of only two rows of cells between Rspl and IR2. Also the patterns of cells between RP1 and IR1 are different in the two fossils. Thus it corresponds to a different unnamed species of Libellulidae.

#### Family Aeshnidae Leach, 1815

##### Aeshna dido

Hagen, 1863

###### Remarks.

This species is based on larval stages. Hagen (1863) placed the specimens in the recent genus *Aeshna* but the available characters are insufficient for this attribution and this taxon must be treated as an Aeshnidae *incertae sedis*, as already indicated in Nel et al. (1994).

### Suborder Zygoptera Selys, 1854

**Family Coenagrionidae Kirby, 1890**

Subfamily and genus undetermined

**Coenagrionidae species A**

Figs 8–10

**Material examined.** GPIBo KH-1a, b from the locality of Rott (Upper Oligocene, sapropelite- and diatomite-layers): nearly complete forwing; body and appendages missing.

**Description.** A nearly complete wing, 16.0 mm long, 3.6 mm wide; distance from wing base to arculus 3.1 mm, from arculus to nodus 2.5 mm, from nodus to pterostigma 8.0 mm, from pterostigma to wing apex 1.4 mm; arculus aligned with Ax2, distance between Ax1 and Ax2 1.2 mm, CuP 0.4 mm distal of base of AA; basal side of discoidal cell 0.2 mm long, costal side 0.5 mm long, distal side 0.6 mm long, posterior side 1.0 mm long; pterostigma 1.1 mm long, 0.8 mm wide, covering one cell, pterostigmal brace pronounced but no distinct angle on RP1 at its level; nine postnodal cross-veins; base of RP2 three cells distal of subnodus; base of IR1 two cells basal of pterostigmal brace; IR2, MA and CuA distally zigzagged, other longitudinal veins nearly straight.

**Discussion.** This fossil wing corresponds to that of a Coenagrionidae for the shape of pterostigma, pronounced pterostigmal brace, relative position of IR2, RP3/4 and subnodus, long petiole, alignment of cross-veins, veins IR2, MA and CuA distally zigzagged, etc. Its exact position within this family is much more delicate to establish on the sole basis of the wing characters. The subfamily divisions proposed by Fraser (1957) or Davies and Tobin (1984) have been recently rejected by O’Grady and May (2003) or Carle et al. (2008). These recent phylogenetic analyses were based on molecular and morphological characters different from the wing venation. The subfamily divisions of the Coenagrionidae are still rather uncertain and need further investigations. Nevertheless this fossil shares with the genera grouped together in the subfamily Argiinae by Fraser (1957) the following characters: CuP well distal of base of AA, discoidal cell distally widened, arculus aligned with Ax2. This wing is very similar to that of the recent genus *Palaiargia* Foerster, 1903 in the position of base of RP2, but it differs from it in the shorter IR1 (Münz 1919). Nel and Papazian (1990) described some Coenagrionidae from the Oligocene of the South of France without naming them. Two of them ‘Argiinae, genre incertae sedis, espèce A’ and ‘Argiinae, genre incertae sedis, espèce B’ were tentatively attributed to the ‘Argiinae’ for the same reasons as above. The new fossil differs from both of them in the base of vein RP2 four cells distal of subnodus instead of three cells, and CuA less zigzagged in ‘espèce A’ and more zigzagged in ‘espèce B’ than in our fossil. Thus the present fossil probably corresponds to a new different species, but naming it is for the present improper. Hagen (1863) described another putative Coenagrionidae from Rott under the name ‘*Agrion icarus*’, transferred by Scudder (1890) in *Platycnemis* but by Kirby (1890) in *Coenagrion*. Its exact affinities remain uncertain (Nel and Papazian 1990; Nel and Paicheler 1993a). It differs from the here described fossil in the greater distance between subnodus and base of RP2, with six cells between them.

## Systematic palaeoichnology

### Ichnofamily Paleoovoididae Vasilenko, 2005

#### Ichnogenus Paleoovoidus

Vasilenko, 2005

* Paleoovoidus Vasilenko, 2005, p. 630, figs 1–3.

part. *Sertoveon* Krassilov, 2008, p. 69, figs 1–5.

*Paleoovoidus* Vasilenko, 2008, p. 516, fig. 2, pl. 7.

*Paleoovoidus* Sarzetti et al., 2009, p. 433, figs 2–7.

“Flea beetle egg deposition,” Lewis and Carroll, 1991, p. 335, fig. 2.

“Flea beetle egg deposition,” Lewis and Carroll, 1992, p. 3, fig. 2.

##### Type ichnospecies.

*Paleoovoidus rectus* Vasilenko, 2005, by monotypy.

##### Diagnosis 

(taken from Sarzetti et al. 2009). Medium-sized elongate, narrow, ovoid, or lens-shaped structures, characterized by regular arrangement in leaf lamina. These structures, defined by dark, surrounding reaction tissue, are narrow at one end, each of which often bears a dark spot.

##### Comments.

Sarzetti et al. (2009) provided a revision of the ichnogenus *Paleoovoidus* including *Paleoovoidus rectus*, *Paleoovoidus flabellatus*, *Paleoovoidus arcuatus*, and *Paleoovoidus bifurcatus*. We have used here the narrowed concept of *Paleoovoidus* as presented by Sarzetti et al. (2009: 437). Other ichnogenera erected by Krassilov and Silantieva (2008), *Costoveon* and *Catenoveon*, are comparable with *Paleoovoidus*, although the disposition of the scars over the leaf are different (*vide* Sarzetti et al. 2009). Unfortunately, the use of nomenclature to describe fossil damage types within a fossil assemblage in the literature is unsatisfactory, not least because of the inconsistency created by different authors using their own particular schemes (e.g., Krassilov and Silantieva 2008; Vasilenko 2008).

#### Paleoovoidus rectus

Vasilenko, 2005

http://species-id.net/wiki/Paleoovoidus_rectus

Figs 11–12

* *Paleoovoidus rectus* Vasilenko, 2005, p. 631, figs 1–3, pl. 5.

“Odonata eggs” van Konijnenburg-van Cittert and Schmeißner, 1999, p. 217.

“Egg scars” Krassilov et al., 2007, p. 806, fig. 3D.

*Paleoovoidus rectus* Sarzetti et al., 2009, p. 437, figs 2.3–2.4.

##### Diagnosis

(taken from Sarzetti et al. 2009: 437). Elongate to lens-shaped scars oriented in a single, linear row, with long axes of scars aligned lengthwise, mostly parallel to the long axis of the leaf and usually occurring along the midrib.

##### Description.

**Figures 11–23.**
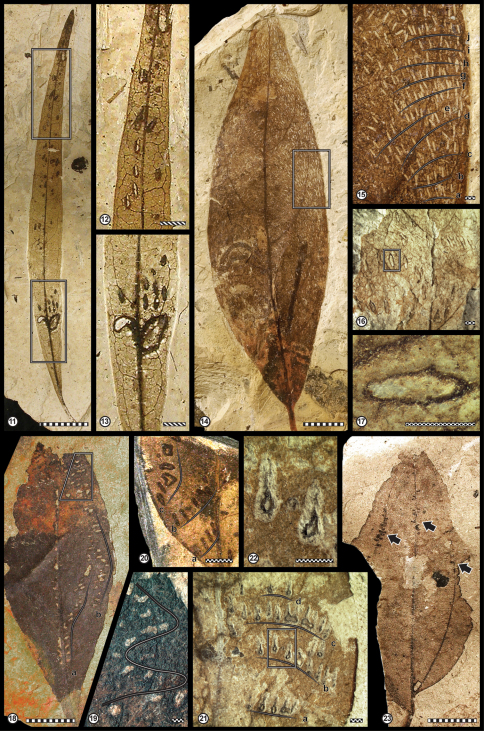
Endophytic oviposition from the Upper Oligocene Fossillagerstätte Rott. *Paleoovoidus rectus* isp **11–12** On *Sideroxylon salicites* (HW_Ro_2.8; Sapotaceae). *Paleoovoidus arcuatus* isp **13** On *Sideroxylon salicites* (HW_Ro_2.8; Sapotaceae), showing a zigzag pattern **14–15** On *Laurophyllum pseudoprinceps* (Ro_10982; Lauraceae), entire leaf fossil showing the distributions of scars over the lamina. Lettered lines (a-j) point to individual rows or ‘files’ of oviposition marks. **16** Trace-fossil specimens GPIBo_Rott_HELL_852 on an indeterminate dicot leaf **17** Enlargement from rectangular template in Figure 16, showing details of an individual scar **18** Entire leaf fossil showing the distributions of scars over the lamina on an indeterminate dicot leaf (Ro_11887). Lettered lines (a-b) point to individual rows of oviposition marks **19** Enlargement from trapezoid template in Figure 18, showing a zigzag pattern **20** Specimen Ro_10355 (an indeterminate dicot leaf) a-c (lettered lines) point to individual rows with a consecutive and parallel pattern **21** Trace-fossil specimens *Apocynophyllum* sp. (GPIBo_Rott_HELL_854, Apocynaceae). Lettered lines (a-d) point to individual rows of oviposition marks oriented along the secondary venation **22** Enlargement from rectangular template in Figure 21, showing teardrop-shaped oviposition scars. *Paleoovoidus bifurcatus* isp **23** On *Zizyphus zizyphoides* (HW_Ro_58.2; Rhamnaceae). Arrows pointing to oviposition scars forming double rows located in an acute angle along to both sides of the veins. Scale bars: stippled bar, 1 cm; slashed bar, 2 mm; dotted bar, 1 mm.

The specimen of *Paleoovoidus rectus* occurs in a leaf of *Sideroxylon salicites* (Sapotaceae, Ro_2.8; Figs 11–12). This leaf has two sets of leaf scars; the inset box in Fig. 11 indicates those corresponding to *Paleoovoidus rectus*. There are seven scars arranged rectilinearly near the leaf apex, aligned closely adjacent along the primary vein, with the scar long axis parallel to the primary vein. The fifth scar is arranged symmetrically about the midvein. All scars show an elongate- to lens-shaped structure with an enveloping raised rim and a central depression (“elongated hole”), indicating the absence of plant tissue.The individual length of the scars ranges from 0.9 to 1.3 mm, and the width ranges from 0.4 to 0.5 mm. The distance between adjacent scars varies from 0.6 to 2.4 mm. Additionally, *Paleoovoidus arcuatus* (Fig. 13) appears basal to the *Paleoovoidus rectus* trace, indicating that both patterns can occur on the same leaf (*vide infra*).

##### Comments.

The specimen of *Paleoovoidus rectus* (HW_Ro_2.8; on *Sideroxylon salicites* [Sapotaceae]) derives from the pelite and lignite facies of the ‘Hangendschichten’ at the Rott locality. The sediments belong to the younger part of the Upper Oligocene (Chattian), based on the mammal assemblage (MP30) recorded by Mörs (1995) with an age of approximately 25 million years as accepted for the Rott Formation (von Koenigswald et al. 1996).

Patterns similar to *Paleoovoidus rectus* described here occur so far only in the early Eocene of Patagonia (Sarzetti et al. 2009) and have a sporadic occurrence on Mesozoic Coniferales and Ginkgoales (Vasilenko 2005; van Konijnenburg-van Cittert and Schmeißner 1999). The Rott specimen exhibits only minor differences from the Argentinian material described by Sarzetti et al. (2009), mainly in dimensions and the total amount of scars preserved. Interestingly, *Paleoovoidus rectus* from the Upper Oligocene occurs at the same position at the tip of the leaf as preserved from the early Eocene Laguna del Hunco floras of Patagonia, and is also associated with *Paleoovoidus arcuatus*.

#### Paleoovoidus arcuatus

(Krassilov, 2008)

http://species-id.net/wiki/Paleoovoidus_arcuatus

Figs 13–22

* *Sertoveon arcuatum* Krassilov, 2008, p. 69, fig. 5.

“Concentric oviposition tracks“ Hellmund, 1986, p. 166 fig. 74; Hellmund, 1987, p. 154, fig. 15; Hellmund, 1988, p. 323.

“Coenagrioniden-Typ“ Hellmund and Hellmund, 1991, p. 7, figs 3.1–3.4, p. 8, fig. 4, p. 9, figs 5.1–5.2; Hellmund and Hellmund, 1993, p. 349, fig. 1, p. 350, fig. 2–3; Hellmund and Hellmund, 1996a, p. 59, fig. 6.3; Hellmund and Hellmund, 1996b, p. 166, fig. 17; Hellmund and Hellmund, 1996c, p. 109, figs 1a, b; Hellmund and Hellmund, 2002c, p. 262, fig. 8a

“Coenagrioniden-Typ vom Bogenmodus“ Hellmund and Hellmund, 1998, p. 282, fig. 1; Hellmund and Hellmund, 2002a, p. 3, fig. 2, p. 10, fig. 8; Hellmund and Hellmund, 2002c, p. 255, fig. 1a, p. 259, fig. 5a, p. 260, fig. 6, p. 261, fig. 7, p. 264, fig. 11a, p. 265, fig. 14, 15.

“Concentric oviposition tracks“ Labandeira, 2002, p. 41.

“Radially oriented oviposition scars“ Labandeira et al., 2002, p. 312, fig. 80.

“Ovoposiciones de la Familia Coenagrionidae“ Peñalver and Delclòs, 2004, p. 74, fig. 2.

“Zygopteran egg sets“ Krassilov et al., 2007, p. 806, fig. 3a–c.

“Endophytic oviposition probably of Calopterygina“ Vasilenko and Rasnitsyn, 2007, p. 1156, figs 4–6.

*Paleoovoidus arcuatus*, Vasilenko 2008 (new syn.), p. 516, fig. 2c, pl. 7, figs 2, 3.[meeting the requirements of ICZN, 1999: Art. 31.2]

*Paleoovoidus arcuatum*, Sarzetti et al., 2009, p. 438, figs 3, 4, 5.1, 5.4–5.6, 6, 7.

*Paleoovoidus arcuatum*, Wappler, 2010, p. 545, figs 3k–l.

##### Diagnosis

(taken from Sarzetti et al. 2009: 438). Elongate, lens-shaped to teardrop-shaped scars arranged with the short axes aligned horizontally to each other, either as straight rows or as arcs. Frequently the long axes of scars are sub parallel to each other. Occasionally, successive rows are parallel or exhibit zigzag patterns.

##### Description.

The endophytic oviposition scars of *Paleoovoidus arcuatus* are quite variable in shape as recognized by Hellmund and Hellmund (1991), Krassilov et al. (2007), and Sarzetti et al. (2009). They range from elongate (Figs 13, 15–17), lens-shaped (Fig. 19), teardrop-shaped (Fig. 22), to more or less irregular shapes (Fig. 20). At the first glance, the scar patterns look chaotic (Fig. 12), but in most cases the scars are arranged in concentric arches (Figs 16, 20–21), but even more or less linear oviposition arcs are realized (Fig. 18). In other cases (Figs 18–19) the arcuate rows are not parallel to each other, resulting in a zigzag pattern (Fig. 19). In general, the dimension of the scars ranges from 1.2 to 1.7 mm in length, and widths range from 0.3 to 0.5 mm. In two cases the specimens cover nearly the entire width of the leaf-blade, containing 111 and 376 scars (Fig. 1.4; comp. Hellmund and Hellmund 1991: Fig 3.1, Hellmund and Hellund 1996a: Fig. 6.3). The scars in rows are usually ranging between 2 to 15. They are variously orientated within the leaves, with the long axis parallel to sub parallel to the primary veins (Figs 16, 20). In some specimens the scars show a distinctive enlargement of the callus (Figs 17, 20, 22).

##### Comments.

The specimens of *Paleoovoidus arcuatus* occur on *Laurophyllum pseudoprinceps* (Ro_10982; Lauraceae), *Apocynophyllum* sp. (GPIBo_Rott_HELL_854, Apocynaceae), *Sideroxylon salicites* (HW_Ro_2.8; Sapotaceae), and on three undetermined dicotyledon leaves (Ro_10355; Ro_11887; GPIBo_Rott_HELL_852; GPIBo_Rott_HELL_851a+b). All specimens derive from the pelite and lignite facies of the ‘Hangendschichten’ at the Rott locality. The sediments belong to the younger part of the Upper Oligocene (Chattian), based on the mammal assemblage (MP30) recorded by Mörs (1995) with an age of approximately 25 million years as accepted for the Rott Formation (von Koenigswald et al. 1996). This type of endophytic oviposition behaviour is widely distributed and known from several host plants, indicating that the species producing this ichnofossil are less selective than the Lestoidea for their angiosperm host plants. The specimen MPEF-Pb-1052 of Laguna del Hunco, is suggestive similar to the ovipositions made by *Epiophlebia superstes* and likely attributed to Frenguelliidae (both Epiproctophora) instead of Coenagrionidae as established by Sarzetti et al. (2009: 444, fig. 5.5).

##### Note.

The ichnogenus *Paleoovoidus* Vasilenko, 2005, originally described from the Upper Jurassic–Lower Cretaceous locality of Chernovskie Kopi, Russia, typically comprises arched oviposition scars, with the eggs set in rows at a considerable distance from each other parallel to their long axes. Since then several ichnospecies have been included. However, considerable confusion persists regarding the ichnotaxonomic status and diagnostic features of the ichnospecies. The ichnospecies *Paleoovoidus arcuatus* Vasilenko, 2008 was published several weeks later than *Sertoveonarcuatum* Krassilov, 2008 (type species of ichnogenus *Sertoveon*: Krassilov and Silantieva 2008). Sarzetti et al. (2009: 438, 441) synonymized these two ichnospecies and established the combination “*Paleoovoidus arcuatum* (Krassilov, 2008)”; however, they erroneously indicated “*Paleoovoidus arcuatum*” as new in their abstract and figure captions. According to ICZN (1999: Art. 31.2), this species name, as a Latin adjective in the nominative singular, must agree in gender with the generic name with which it is at any time combined, therefore is here corrected to: *Paleoovoidus arcuatus* (Krassilov, 2008).

#### Paleoovoidus bifurcatus

Sarzetti, Labandeira, Muzón, Wilf, Cúneo, Johnson & Genise, 2009

http://species-id.net/wiki/Paleoovoidus_bifurcatus

Fig. 23

* *Paleoovoidus bifurcatus* Sarzetti et al., 2009, p. 438, figs 2.1, 2.2.

“Galle *Aceria nervesqua fagina*“ Straus, 1977, p. 74, fig. 2, p. 78, fig. 50.

“Oviposition damage on primary and secondary veins (“Doppelreihen Modus“)“ Hellmund, 1988, p. 323.

“Lestiden-Typ“ Hellmund and Hellmund, 1991, p. 4–5 figs 1.1–1.3, 2; Hellmund and Hellmund, 1996a, p. 58, fig. 6.1a–b; Hellmund and Hellmund, 1996b, p. 165, fig. 16; Hellmund and Hellmund, 2002a, p. 3, fig. 2; Hellmund and Hellmund, 2002b, p. 49, figs 2–3, p. 53, fig. 12.

“Oviposition damage on secondary veins“ Labandeira et al., 2007, p. 10.

##### Diagnosis

(taken from Sarzetti et al. 2009: 438). Elongate to lens-shaped scars arranged in pairs along both sides of a primary vein, forming double rows and sometimes a V-shaped configuration, with the arms of the V parallel to secondary veins and the vertex embedded in the midvein.

##### Description.

The oviposition scars are preserved on a nearly complete preserved lanceolate leaf. Base acute and slightly asymmetric. Venation imperfect basal acrodromous.Midrib moderately thick and straight. Besides the pair of stout secondary veins arising at the base, secondary and tertiary veins form a fine network. The ovoid or ellipsoidal-shaped oviposition scars occur in pairs along the midrib and oriented at a right angle with respect to the vein in the upper part of the leaf. Main cluster occurs on the secondary vein, whereas the occurrence of endophytic oviposition scars on the midrib vein is more scattered. The total amount of scars is 25. The axial length of the scars ranges from 0.5 mm to 0.8 mm, and their width ranges from 0.3 mm to 0.4 mm. The distances between consecutive scars are variable within the range of 0.5 mm to 0.7 mm.

##### Comments.

The specimen of *Paleoovoidus bifurcatus* (HW_Ro_58.2, on *Zizyphus zizyphoides* [Rhamnaceae]) derives from the pelite and lignite facies of the ‘Hangendschichten’ at the Rott locality. The sediments belong to the younger part of the Upper Oligocene (Chattian), based on the mammal assemblage (MP30) recorded by Mörs (1995) with an age of approximately 25 million years as accepted for the Rott Formation (von Koenigswald et al. 1996). The pattern was originally mentioned by (Hellmund (1986, 1987, 1988) and originally described and figured by Hellmund and Hellmund (1991, figs 1–2) but without any ichnotaxonomic analyses. A new ichnospecies, *Paleoovoidus bifurcatus*, was described by Sarzetti et al. (2009) from the early Eocene Laguna del Hunco floras of Patagonia. Accordingly, the ichnotaxonomic status of the ichnogenus *Paleoovoidus* was reviewed, providing a new ichnotaxonomic classification, indicating that the preservation of *Paleoovoidus bifurcatus* is variable across a broad range of compression/impression floras and host plants. The presense of *Paleoovoidus bifurcatus* on the Buckthorn family (Rhamnaceae) at Rott extending its host plant range. Previously, the ichnogenus show a marked preference for lauraceous leaves of the morphogenus *Daphnogene cinnamomifolia* (Hellmund and Hellmund 1991, 1996a, b).

## Discussion

In Rott the two main modern lineages of Odonata, Zygoptera and Anisoptera, are recorded. Additionally, the Sieblosiidae, a basal and extinct epiproctophoran family (Nel et al. 2005), is also recorded there. This family is only present in the Eocene to the Miocene of Europe. The Epiproctophora nec Anisoptera are mainly Mesozoic, while the Cenozoic to Recent representatives of this grade are the Sieblosiidae, the Eocene Frenguelliidae from Patagonia (Petrulevičius and Nel 2003, 2007), plus the recent Epiophlebiidae present in Japan and Himalaya. Latter family, with two species, is the unique survivor of these damsel-dragonflies. With respect to the structure of their functional ovipositors, the Sieblosiidae were probably laying their eggs in aquatic plants and/or floating leaves but their type of oviposition remain unknown. Despite their damselfly-like habitus of the adults, the Sieblosiidae are distinctly larger than the Coenagrionoidea and Lestoidea already known from Rott, suggesting that their eggs and oviposition traces could have been also larger. Anyway, the presence of a zigzag modal of oviposition in Rott and Laguna del Hunco similar to that of an Epiophlebiidae is suggestive but could as well have been caused by an aeshnid, also recorded in Rott.

As supposed, the diversity of traces of oviposition left on the leaves together with the presence of larvae in the fossil record from Rott indicate that many of the Odonata found as adults in this outcrop were autochthonous. This is not the case for the nearly contemporaneous outcrop of Céreste in which adult Odonata are frequent and diverse but larvae and trace of oviposition are still unrecorded (Nel pers. obs.). The impressive rarity of aquatic beetles (only one known specimen of Hydrophilidae for more than 60000 fossil insects, Nel collection) at Céreste also greatly contrasts from their diversity at Rott (Fikáček et al. 2010). These data are of interest for a better estimation and comparison of the former quality of lake water and general palaeoecological reconstructions of these localities.

## Supplementary Material

XML Treatment for Libellula cassandra

XML Treatment for Libellula ceres

XML Treatment for Libellulidae

XML Treatment for Aeshna dido

XML Treatment for Paleoovoidus

XML Treatment for Paleoovoidus rectus

XML Treatment for Paleoovoidus arcuatus

XML Treatment for Paleoovoidus bifurcatus
